# Increasing mixed-berry flavonoid intake may reduce depressive symptoms in older adults: Framingham Heart Study

**DOI:** 10.1093/geroni/igab046.3132

**Published:** 2021-12-17

**Authors:** Courtney Millar, Alyssa Dufour, Marian Hannan, Shivani Sahni

**Affiliations:** 1 Hebrew SeniorLife, Roslindale, Massachusetts, United States; 2 Hebrew SeniorLife, Boston, Massachusetts, United States

## Abstract

Depression affects more than 250 million people worldwide. Although epidemiological studies have linked higher dietary flavonoids with depression prevention in older women, it is unknown if increasing dietary flavonoids could effectively reduce depression. Mixed berries (blueberry, blackberry, and raspberry) are a rich source of flavonoids, particularly anthocyanin, flavanol, and flavan-3-ol subclasses. Our aim was to determine the association of mixed-berry flavonoid intake with change in depressive symptoms over ~8 years in older adults from the Framingham Heart Study. This community-based prospective longitudinal study included 1,278 adults with assessments on diet (food frequency questionnaire) and depressive symptoms (Center for Epidemiologic Studies Depression, CES-D) at baseline (1998-2001) and follow-up (2005-2008). Absolute change in mixed-berry flavonoid intake (defined as sum of anthocyanin, flavanol, and flavon-3-ols, mg/day) and change in CES-D scores were calculated. Linear regression estimated beta and standard error (SE) for change in CES-D scores per 250 mg/day increase in mixed-berry flavonoids (obtained from ~3/4 cup of mixed berries), adjusting for baseline age, sex, energy-intake, current smoking, body mass index, physical activity, cardiovascular disease, and non-melanoma cancer. Mean age was 59±9 years (range: 33-81), 57% female and mean change in mixed-berry flavonoid intake was 15.0±72.8 mg/day over ~8 years. In adjusted models, each 250 mg/day increase in mixed-berry flavonoid intake was associated with a 1-point reduction in depressive symptoms (beta: -1.06, SE: 0.61, p=0.08) over ~8 years, although this was not statistically significant. These data highlight the need for randomized clinical trials of flavonoid-rich berries to target depressive symptoms in older adults.

